# Developing physiotherapy student safety skills in readiness for clinical placement using standardised patients compared with peer-role play: a pilot non-randomised controlled trial

**DOI:** 10.1186/s12909-017-0973-5

**Published:** 2017-08-10

**Authors:** Anna C. Phillips, Shylie F. Mackintosh, Alison Bell, Kylie N. Johnston

**Affiliations:** 1 School of Health Sciences, University of South Australia, GPO box 2471, Adelaide, 5001 Australia; 2 Alliance for Research in Exercise, Nutrition and Activity (ARENA), Sansom Institute for Health Research, University of South Australia, GPO Box 2471, Adelaide, 5001 Australia

**Keywords:** Simulation, Standardised patients, Physiotherapy, Safety, Pilot trial

## Abstract

**Background:**

Using simulated learning environments with standardised patients (SPs) provides a way to scaffold the development of skills for patient safety in a low risk environment. There are no data regarding whether adding SP interactions in early years of physiotherapy training improves safe performance on clinical placement. We assessed the feasibility of recruiting and collecting data from junior physiotherapy students during an SP workshop with a pilot non-randomised trial, also assessing time, cost and scheduling information.

**Methods:**

Second year physiotherapy students were invited to participate and allocated to either the SP workshop in a simulated hospital environment (with and without video feedback) or usual teaching comprising peer role play. The main outcome measures were participant recruitment, retention and survey response rates, whether the training and workshops were delivered as scheduled and costs for SPs and staff training and workshop attendance. Students self-reported confidence, communication, preparedness for clinic and satisfaction was measured using pre-post surveys.

**Results:**

The pilot trial proved feasible, with 108 students recruited (100%) and high retention (95%) and survey response rates (85%). The training sessions and SP workshops were delivered as scheduled, costing $4700AUD. Students rated their confidence and preparedness for clinical placement higher post intervention (*p* < 0.001) with high levels of satisfaction with the SP interactions (mean score 9.3/10).

**Conclusions:**

In this setting the SP workshop was feasible. Further research incorporating a randomised trial investigating the integration of SPs for the development and assessment of patient safety skills in physiotherapy education is recommended.

**Trial registration:**

ANZCTR no: 12,615,000,686,505.

**Electronic supplementary material:**

The online version of this article (doi:10.1186/s12909-017-0973-5) contains supplementary material, which is available to authorized users.

## Background

Safe practice is essential to health professional competency. Australian higher education providers offering physiotherapy programs must ensure graduates meet the threshold competencies required for professional registration reflecting safe, independent and effective physiotherapy practice across a range of contexts and settings [1, 2].

University-based teaching and learning strategies aim to develop the foundational abilities of knowledge, skills, attitudes, values and judgements for competent physiotherapy practice. Classroom-based learning includes practice of assessment and techniques with student peers in role-play scenarios. Supervised clinical placements then provide experiential learning where students gain concrete experience, and have opportunities for observation and reflection, formation of abstract concepts and practising new skills [3]. Clinical placements in the latter stages of physiotherapy programs are the setting for assessment of competency standards [4].

Significant preparation is required for clinical placements to develop students’ capacity to execute safe patient management across community and hospital settings. Developing competencies for safe physiotherapy practice in acute hospital settings poses particular challenges such as complex physical environments, medically unwell patients and organisational requirements including communication, documentation and timeliness. Clinical educators have described the high investment of time and stressful nature of working with students who are struggling with patient safety issues on clinical placement [5].

Knowledge and competencies required to deliver safe patient manual handling in an acute setting encompass communicating effectively; identifying, preventing and managing adverse events and near misses; using evidence and information; working safely as a team; being ethical; and continuing learning [6]. A multiple case study of eight UK medical, nursing and allied health undergraduate programs reported that patient safety was viewed as implicit in the curricula as an overall program outcome [7]. Supervised practice in the clinical setting was seen as pivotal in the development of safety skills. However, the gap between university-based teaching and clinical practice was recognised, as in this statement by a third year physiotherapy student:

*“..we do our assessments and our practical things like (…) keeping the patients safe but you don’t actually learn it until you’re out on placement, until you are in that setting..* [7]”

A potential strategy to scaffold the development of skills for patient safety is the use of simulated learning environments with standardised patient (SP) scenarios. This strategy uses individuals trained to present as a patient with a specific condition, combined with a simulated clinical environment. This approach has been evaluated, with positive results, in the preparation of physiotherapy students for clinical placements, or to replace part of clinical time [8–12]. Standardised patient scenarios allow students to practise assessment, clinical decision making and intervention in a safe environment that is supportive, controlled, low risk and able to be progressed to suit students’ developing skill level. The advantages for students include immediate feedback, the ability to reflect on their practice, and alter practice accordingly without the ethical and safety implications of 'making mistakes' on real patients [8].

As patient mobilisation in an acute setting had previously led to repeated student failure on clinic, we were specifically interested in bridging the gap between manual handling skills for patient mobilisation learned in the foundational physiotherapy courses (first and second years) and development of competencies on clinical placements (third and fourth years). Using SP scenarios presents a means to scaffold student learning, and we sought to evaluate the feasibility of conducting a three-hour practical session using SPs in a clinical scenario [13].

As a means of evaluating the feasibility of introducing SP interactions early in the physiotherapy program, we undertook pilot testing to determine the process and resource requirements for conducting a three hour practical workshop incorporating SPs in a clinical scenario (with and without video feedback) involving a manual handling task to develop manual handling skills. This session was in contrast to usual teaching and learning practice that involved students’ role playing as patients.

Therefore the primary aims of this pilot study were to evaluate (1) feasibility, including the process and resource requirements, of conducting a practical workshop with SPs in a clinical scenario with and without video feedback; (2) participant satisfaction with the SP interactions, and ratings of confidence, perceived preparedness for clinical placement; (3) whether optional video feedback impacted on student confidence, perceived preparedness for clinical placement and (4) whether any differences were observed in OSCE scores, number of student fail grades between students interacting with SP scenarios compared with usual peer role play scenarios.

## Methods

### Design

This pilot study used a non-randomised controlled design. The study conditions were either (A) SP scenario in simulated hospital environment with SP feedback only; (B) SP scenario in a simulated hospital environment with SP and optional video feedback; or (C) peer role play of the patient scenario in a usual practical classroom.

### Participants

All second year undergraduate physiotherapy students enrolled in the relevant physiotherapy course at the University of South Australia were invited to participate in this study.

### Procedure

Students were allocated by usual university processes to one of three practical groups prior to the invitation to participate in the study. Each practical group was allocated a study condition (A,B,C) by a team member not involved with usual teaching of this course and blind to student identity or characteristics (KJ).

Practical sessions were 3 h in duration (identical for each study condition) and held sequentially over 1 day. In all study conditions participants worked in small groups (3 or 4) and were facilitated by one physiotherapy clinical educator.

The educators involved in the workshop sessions (*n* = 6, 1 male) were experienced clinical educators and attended a one-hour training session 2 weeks prior to the intervention.

#### Condition A: Standardised patient scenario session without video feedback

A comprehensive, standardised patient scenario was developed by the research team and reviewed and refined by a panel including external experts. The scenario involved physiotherapy assessment and assistance to mobilise out of bed for an older woman after surgical management of a hip fracture. Student learning objectives for the session were developed in line with course objectives and integration of experiential learning theory (Additional file 1) [3].

A patient scenario guide (including script and photographs of the standardised patient) was developed (Additional file 2). To promote standardisation of the patient scenario, all five female standardised patients attended a two-hour training workshop led by a physiotherapy educator with expertise in training standardised patients.

The learning activity included two standardised patient encounters each comprising 30 min preparatory time, 30 min for patient interaction and 30 min for debriefing immediately following the patient encounter (Additional file 3). A debrief script was used to facilitate standardised debrief sessions (Additional file 4).

Students were provided with standardised patient information (Additional file 5) and support from a clinical educator during the preparation time (30 min). During the 30 min patient interaction, each group of four student participants worked in pairs, one pair as therapists (lead and assistant) and the other pair as peer observers. After 15 min the pairs switched roles. Each group was supervised by a clinical educator. The debrief session, facilitated by a clinical educator, guided by the debrief script, focussed on student self-reflection of their performance and peer feedback (30 min). After debriefing, participants prepared for and practiced the second patient scenario (30 min). The second patient encounter incorporated a randomised safety issue during the patient response to standing and walking (i.e. becoming light-headed, nausea, or feeling weak in the legs). This was followed by a final debriefing session (30 mins).

#### Condition B: Standardised patient scenario session with optional video feedback

This study condition was identical to Intervention A, with the addition of video recording of each patient interaction by the student peer observers. During the debrief session the participants were invited to watch the video replay of their own performance in addition to self-reflection and peer feedback. Video files were deleted at the completion of each debrief session.

#### Condition C: Peer role play scenario session

Participants worked in groups of four to role-play the same case scenario. As this followed usual teaching practice, the use of a simulated environment was not included (plinth not hospital bed, no attachments), and students took on the role of the patient. The sequence and timing of preparation, patient interaction/role play and debriefing were similar to the intervention conditions. Over the course of the practical session the participants rotated through the roles of ‘patient’, ‘lead therapist’, ‘assistant’ and ‘observer’. At the completion of each patient interaction, small group debrief was conducted, facilitated by an educator, incorporating peer and facilitator feedback and the opportunity for self-reflection by the participants.

## Outcome measures

### Feasibility

Feasibility for this pilot trial was based on the broad classifications recommended by Thabane et al. [13], focussing on the process and resource requirements for the workshops. The process requirements evaluated were participant recruitment, retention and survey response rates and whether the training for clinical educators and SPs and workshop sessions for SP interactions including the debriefing and feedback (from the SP and video) were able to be delivered as planned. The resource requirements evaluated were the total time taken and costs ($AUD) associated with the training for and participation in the workshops for both SPs and clinical educators.

### Surveys

A survey developed by Mandrusiak et al. [8], was adapted with permission and administered to the SP intervention groups. Ten statements that covered communication, confidence, preparedness for placement and self-perception of their communication, practical and clinical reasoning skills were presented to the participants before and after the SP interactive experience. Further statements (*n* = 10) were presented to the participants after the session that explored qualities of the learning experience including student motivation and interest, value of feedback from the SP, debrief sessions and realism. Participants were asked to respond to each statement by marking a visual analogue scale between zero (strongly disagree) and 10 (strongly agree). Participants were also invited to provide written comments in response to two questions:

1. *How was this role playing helpful?*
2. *How could this experience be improved?*

### Practical examination mark

As part of usual course requirements, all participants were assessed in an objective structured clinical examination (OSCE) on skills relevant to safe mobilisation of a patient including assessment and manual handling. This took place at the end of semester, 5 weeks after the study intervention workshop day. In this 10 min assessment, conducted in a university practical room, the student was presented with a brief case study and task involving mobilisation. The student was asked to demonstrate the task and a staff member played the role of the patient. The assessment criteria were not altered for this study with allocation of marks for clinical reasoning (30%), set-up safety of the environment, therapist and patient (30%), and appropriate execution throughout task including monitoring (40%); total score for this component was ten marks. Examiners of the OSCE were blinded to student group allocation.

The number of students assigned a fail grade was recorded. A fail grade was assigned for an OSCE score of less than five or where a breach of safe or professional practice occurred such as leaving the bed brakes off or failure to comply with infection control procedures.

## Data analysis

### Analysis

Descriptive statistics were used to describe and explore the feasibility measures, participants’ characteristics, survey responses and OSCE scores. Pre-post workshop changes in perceived confidence, perceived preparedness for clinical placement and satisfaction with the use of the SP scenarios were examined within the intervention groups for each of the ten items of the survey using paired t-tests. Averages of OSCE scores and number of fails observed for students in each of the study groups are reported but due to non-random allocation have not been statistically compared. The software package SPSS v17.0 (SPSS Inc., Chicago, IL, USA) was used for all analyses.

Written comments provided to open ended questions by participants were de-identified, extracted verbatim and collated. Comments were reviewed and those with common content grouped into major and minor categories (AP) [14]. Organisation of extracts into categories was discussed with a second researcher (KJ) until consensus was reached and a descriptive summary generated including examples of data excerpts to illustrate the categories.

## Results

### Feasibility

#### Process

Recruitment for this study was 100%, with all 108 potential participants enrolled in the practical classes providing written informed consent to participate in the study. One hundred and three participants attended the workshop sessions (95% uptake). The response rate for the surveys was 85%, with 56 of 66 participants submitting complete pre-post surveys. All preparatory training sessions for the clinical educators and SPs were delivered as planned, and all student-SP interactions, feedback and debrief sessions were delivered as scheduled on the workshop day. As video feedback was optional, many students chose not to observe their recorded performance, however no issues were encountered with the recording or playback for the video feedback.

#### Resources

The direct cost of running the workshop was $4700 AUD ($71.21per student), comprising costs of $2250 for SPs and $2450 for clinical educators. The time spent for training and delivery for the SP workshops was approximately 80 h, comprising 20 h for pre-intervention training (15 h SPs, 5 h clinical educators) and 60 h for the intervention (30 h each SP and clinical educator time). The direct cost of running the usual teaching was $900AUD ($24.32 per student) for 12 h of time for two clinical educators (6 h preparation, 6 h clinical education).

### Participants

**Fig. 1:**
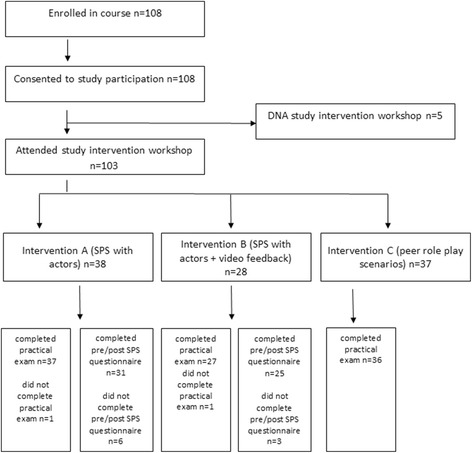
Shows the participation flow through study interventions and completion of outcome measures. Legend DNA = did not attend; SPS = standardised patient scenario

All 108 potential participants enrolled in the practical classes gave written informed consent to participate in the study. One hundred and three participants attended the workshop. Complete data for participants in the SP workshops were provided by 56 (85%) participants (Fig. 1). No adverse events were encountered.

Participant characteristics are shown in Table 1.

**Table 1:** Characteristics of study participants

	All participants *n* = 103	Intervention A SPS + SP feedback *n* = 38	Intervention B SPS + SP feedback + optional video feedback *n* = 28	Intervention C Peer role play scenarios *n* = 37
Age in years median(IQR)	19(2)	19(1)	20(2)	19(4)
Male gender n(%)	42(41)	12(32)	16(57)	14(38)
Grade point average (GPA) (scale out of 7) mean(SD)	5.76(0.69)	5.95(0.59)	5.48(0.73)	5.79(0.70)

*SP* standardised patient, *SPS* standardised patient scenarios

For all participants in the SP interactions, this was their first exposure to SPs. Baseline characteristics differed between the three groups with regard to median age, gender proportion and baseline GPA. Intervention B included the greatest proportion of male students, and had the lowest GPA [mean (SD) 5.48(73)] (Table 1).

## Survey responses

### Preparedness for clinical placement

Participants in the standardised patient scenario groups (Interventions A and B, *n* = 57) rated all 10 survey statements about their perceived self-efficacy significantly higher post-intervention compared with pre-intervention (*p* < 0.001) (Table 2).

**Table 2:** Summary of participants’ pre-post survey ratings regarding their perceived preparedness for clinical placement

Survey statement		Pre experience *n* = 57 mean(SD)	Post experience *n* = 57 mean(SD)	Mean change (95% CI)	*p* value
1	I feel confident in my ability to complete this activity to a high standard	4.0(1.8)	6.7(1.4)	2.6 (2.1 to 3.2)	*p* < 0.001
2	I feel prepared for clinical placement in the area of physiotherapy associated with this role playing	3.4(1.8)	6.1(2.1)	2.6 (2.2 to 3.1)	*p* < 0.001
3	I am aware of my strengths in this role playing activity	4.6(1.6)	7.6(1.5)	3.0 (2.6 to 3.5)	*p* < 0.001
4	I can identify areas of weakness related to this activity where I would benefit from further preparation for clinical placement	6.8(1.6)	8.3(1.6)	1.5 (0.9 to 2.0)	*p* < 0.001
5	I feel confident in my ability to establish rapport with a client	5.6(2.0) *n* = 55	7.2(1.9) *n* = 55	1.6 (1.1 to 2.1)	*p* < 0.001
6	I feel confident I can use interpersonal skills such as reflective listening and appropriate use of questions when interacting with real clients	6.0(1.8) *n* = 56	7.4(1.7) *n* = 56	1.4 (0.9 to 1.9)	*p* < 0.001
7	I feel confident that I can provide information and education to clients	5.5(1.8) *n* = 56	6.9(1.6) *n* = 56	1.4 (1.0 to 1.8)	*p* < 0.001
8	I feel confident in my ability to use appropriate handling and practical skills with this client type	4.9(1.9) *n* = 56	6.8(1.8) *n* = 56	1.9 (1.4 to 2.4)	*p* < 0.001
9	I feel confident I can interact in a professional manner	7.0(2.0) *n* = 56	8.2(1.4) *n* = 56	1.2 (0.7 to 1.7)	*p* <0 .001
10	I feel confident I can identify key problems during an assessment	5.6(1.6) *n* = 56	7.1(1.7) *n* = 56	1.5 (1.0 to 2.0)	*p* <0 .001

The areas participants rated as the most improved (mean change >2.5 points on a 10 point scale) were Statement 3 “I am aware of my strengths in this role playing activity” Statement 1 “I feel confident in my ability to complete this activity to a high standard” and Statement 2 “I feel prepared for clinical placement in the area of physiotherapy associated with this role playing” (Table 2). Statement 9 “I feel confident I can interact in a professional manner” achieved the lowest mean change in rating by participants (mean change 1.2, 95% CI 0.7 to 1.7); however, it should be noted that students reported high levels of confidence in this item pre-intervention.

All participants reported very high satisfaction with this learning activity [mean (SD) VAS rating 9.3(0.9)]. The learning experience was also rated very highly [mean (SD) VAS 8.7(1.2)] for ability to motivate and interest students. Highly valued qualities of the experience included realism and feedback from the SPs and the debrief sessions.

### Participant comments

Written comments in response to the first question, "﻿How was this role playing helpful?" (*n* = 57 participant responses), were synthesised in four major categories relating to realism, self-reflection, learning opportunities and practice (Table 3).

**Table 3:** Summary of student responses for “How was this role playing helpful?”

Major category	Minor (sub) category	Frequency of written comment^a^
Realism		33
	Realistic experience	14
	Realistic environment	8
	“Real” patient	7
	Provided context for learning	4
Self-reflection		30
	Identifying strengths	15
	Identifying weaknesses	8
	Problem solving skills	4
	Improved confidence	3
Learning opportunities		24
	From watching others	8
	From feedback	6
	To put it all together	5
	From challenges/complications	3
	Expectations for learning	2
Practice		14
	Manual handling	7
	Managing attachments	4
	Patient management	3

^a^Written comments from some participants included extracts relevant to more than one category

The greatest number of participant comments about helpful aspects of the standardised patient activity related to the realism of the intervention in terms of the experience (*n* = 14) and the environment (*n* = 8). One participant (Participant 2) commented, *“The patient had 'real' symptoms and actually needed assistance which gave me a stronger understanding of what is required by me.”*

Many participants commented that the standardised patient experiences provided an opportunity for self-reflection, relating to the identification of strengths (*n* = 15), weaknesses (*n* = 8), and improved confidence (*n* = 3). For example;

**“** *This has improved my confidence and skills in leaps and bounds from just today’s session. I was extremely uncertain/nervous before this and now feel like I can do this. PLEASE add this to courses in the future. I can't talk about this enough.”* Participant 48

Twenty-four participant comments related to the learning opportunities provided in the intervention sessions. These included watching others (*n* = 8), learning from feedback (*n* = 6) and challenges or complications (*n* = 3). For example;

*“….very different to when we 'role-play' patients even if given a transcript to act, still isn't the same. Was very beneficial even just being able to watch colleagues do the transfer.”* Participant 15

Eleven participants comments related to the helpfulness of the standardised patient experience in the practice of manual handling (*n* = 7) and managing attachments (*n* = 4). For example;

*“..it was helpful to perform the manual handling techniques in a simulated environment. It was stressful and difficult but it really emphasised the importance of safety and the fact that all client presentations are different.”* Participant 18

Comments in response to the second question, "﻿How could this experience be improved?", were summarised into four major categories relating to patient interactions, changes to the interaction, the clinical educator, and preparation for the intervention (Additional file 6).

There were few recommendations for improvement provided by participants, with the majority of comments requesting a greater number of sessions/more exposure to the interactions (*n* = 12), more time in the sessions (*n* = 5), more feedback from the clinical educator (*n* = 5), different types of patient scenarios and more preparation in the days/weeks prior to the intervention (*n* = 4) (Additional file 6).

### OSCE scores

The student OSCE scores (out of 10) are shown in Table 4.

**Table 4:** OSCE score attained and number of student fails

	Intervention A SPS + SP feedback *n* = 37	Intervention B SPS﻿ +﻿ SP feedback + optional video feedback *n* = 27	Intervention C Peer role play scenarios *n* = 37
OSCE score/10 Mean (SD)	7.4(1.9)	5.9(1.8)	7.1(1.6)
Fails in OSCE *n*(%)	5(13.5)	4(15.4)	3 (8.1)
Safety fails in OSCE *n*(%)	5(13.5)	4(15.4)	1(2.7)

*SP* standardised patient, *SPS* standardised patient scenarios, *OSCE* objective structured clinical examination

Data suggests that on average lower OSCE scores were seen for participants in Intervention B than Intervention A, but suggests no differences pairwise between either of the standardised patient scenarios (Intervention﻿ A or B) and the peer role play scenario (Intervention﻿ C). Twelve (12%) students achieved a fail grade in the OSCE exam, with the greatest proportion of student fails in Intervention B (standardised patient scenario) (15.4%) (Table 4).

## Discussion

This pilot study investigated the feasibility of introducing an SP scenario workshop in the early stage (second year) of physiotherapy education on the development of manual handling and patient safety skills.

Evaluation of the workshop demonstrated high feasibility for participant recruitment, participant retention and survey completion (response rates), training for both clinical educators and the SPs and delivery of the workshop proper. All student-SP interactions, debriefing and feedback sessions from peers, SP and/or video were completed as scheduled. A total of 80 h of time was required for preparation and execution of the SP workshop, 20 h training and 60 h for the workshop. The workshop cost $4700 AUD to run.

This SP workshop was more expensive to deliver than usual teaching, at an additional $46.89 per student. However, it is difficult to compare our workshop costs with other SP interventions as information reported for time and costs associated with SPs interventions is scarce [15]. A systematic review by Patricio et al. [15] found that 4% of studies reported information regarding time and costs associated with SPs and only 2% reported information regarding staff time and costs. The costs associated with running this workshop per student, equated to $71.21AUD, while almost three time more expensive than usual teaching, were comparable with the costs of $70CAN per student reported by Poenaru et al. [16] attempting to run an OSCE on a shoe string budget, and considerably less than the direct costs reported for low stakes OSCE’s of $170 to $438USD per student [16]. The relatively low costs for this SP workshop may be considered worthwhile to improve student preparedness for clinical experience, given the high economic costs associated with failure on clinical placement (US$9371 per student failing) [17].

Participants in the standardised patient scenarios were highly satisfied, interested and motivated by this learning experience. There were significant improvements in students’ perceived preparedness for clinical placement: specifically confidence in interpersonal skills, establishing client rapport, identifying key problems, providing education, ability to use appropriate handling and identification of weaknesses and strengths. Effective features of the standardised patient scenarios were highlighted by consistencies across pre-post improvements on VAS scales, post-experience ratings and written comments. For example, the SP scenario experience promoted student self-reflection on performance with a focus on student strengths. This is consistent with reports of increased insight into ability in scenarios where senior physiotherapy students acted as standardised patients for junior students [8]. Self-reflection is a key element in the theoretical basis of Kolb’s experiential learning framework and recognised as critical in the development of physiotherapy practitioner skills [3, 18]. In retrospect, evaluation of participant perspectives and satisfaction with the peer role play scenarios would have been useful and enabled comparisons between the interventions.

Participation in the SP scenarios did not appear to result in any difference in the OSCE scores in the assessment of manual handling skills or the number of student fails in the OSCE compared with the usual peer role play. Although video feedback was proposed to provide additional benefits for participants, this was not reflected in the participant satisfaction, perceived confidence, preparedness for placement or OSCE scores (participants in Intervention B achieved the lowest overall OSCE scores). As the video feedback was optional, it is possible not all participants chose to watch their recorded performance. However, whether any true differences existed between these groups who received the two types of standardised patient scenarios cannot be determined from this pilot study, due to lack of random allocation and baseline difference between groups in GPA, age and gender. Further research incorporating a randomised trial investigating the integration of SPs for the development and assessment of patient safety skills in physiotherapy education is recommended. Future recommendations also include the provision and retention of video feedback beyond the SP interaction, which may provide a valuable learning opportunity for students to view their debriefing files individually at a later stage.

### Limitations

Our pilot study sample was non-randomised, constrained by student enrolment and conducted at a single institution, which limit the interpretation and generalisability of the results. These issues are commonly recognised limitations in medical education research [19]. The survey measure has been previously used to report student self-efficacy and preparedness for placement but does not have established reliability or validity. This survey measure was sought from the SPS groups only and meant that comparison of the student experiences across the intervention and control groups was not possible.

It is likely that this one-off exposure to a SP workshop experience offered limited opportunity to effect change in the skills evaluated in the OSCE in comparison to role-play scenarios. Students recognised the value of manual handling and mobility practice with a realistic patient and-environment and requested more frequent exposures over the curricula, with different client scenarios.

Our study findings therefore offer suggestions for how a SP simulation experience could be integrated in physiotherapy curricula to support the development of skills required for safe patient management. Mori and colleagues [20] have recommended a staged application of simulation learning experiences throughout the physiotherapy curricula [20]. Based on the “challenge point framework” of motor learning described by Guadagnoli and Lee [21], they suggest use of “random” simulation practice for learning and retention of difficult skills [20, 21]. Incremental, coordinated use of simulation amongst other learning and teaching approaches aligns with recommendations from the WHO framework for patient safety curricula (2011), and findings of observational studies in health care education for patient safety [7, 22]. Both call for an integrated approach that make patient safety issues more visible and easier to track throughout the whole education program.

Patient safety frameworks have been largely developed for medical, nursing and pharmacy education and have emphases on invasive procedures and medication safety [22, 23]. For physiotherapists working in hospital settings, skills to manage unwell patients’ safety during mobilisation and physical handling are core competencies [24, 25]. This aspect of patient safety needs further exploration regarding the best way to stage the development of required skills, knowledge and attitudes throughout the undergraduate curricula, including development of valid criteria to assess student learning in relation to patient safety [7].

## Conclusion

This pilot trial provides important information regarding the feasibility of implementing a single SP scenario workshop. The cost of the workshop was $4700 and required 80 h of time for training and participation. Although more expensive than traditional peer role play scenarios, recruitment (100%) and participation (94%) rates were high, as were survey response rates (85%). Training for the SPs and clinical educators was completed successfully and all SP interactions, feedback and debrief sessions were delivered as scheduled. Participants in the SP groups reported improvements in confidence and perceived preparedness for clinical placement and high levels of satisfaction with the SP interactions. These findings can be used to inform future research in optimal ways to integrate larger scale SP workshops for junior physiotherapy students in the development and assessment of patient safety skills during mobilisation and manual handling in physiotherapy student education.

## Additional files

Additional file 1: Feedback checklist and key learning objectives for standardised patient scenario workshop. Description of data: The checklist used by clinical educators during the standardised patient scenario workshops to provide standardised feedback covering the key learning objectives for the workshop. (PDF 291 kb)

Additional file 2: Training information provided to SPs. Description of data: Information provided to the SPs including background information for the patient and the required behaviours, movements and attachments required for the workshop scenarios. (PDF 514 kb)

Additional file 3: Timetable for standardised patient scenario workshop. Description of data: The timetable used for the standardised patient scenario workshop allowing each group of students to experience two patient encounters with preparation and debriefing time for each encounter. (PDF 223 kb)

Additional file 4: Facilitator guide for student debriefing session. Description of data: The guide provided to facilitators to assist the two debriefing sessions immediately following the completion of each of the student encounters with the standardised patient scenarios. (PDF 196 kb)

Additional file 5: Student vignette. Description of data: The information provided to students prior to the standardised patient scenario workshops which included a summary of all relevant patient information and images of the patient’s attachments to assist in the preparation for the patient encounter. (PDF 312 kb)

Additional file 6: Student response provided for how the standardised patient scenarios could be improved. Description of data: A summary of all verbatim responses provided by students following the completion of the standardised patient scenario workshops in response to the question “How could this experience be improved?” (PDF 212 kb)

## Abbreviations

AP: Anna Phillips
AUD: Australian dollars
CAN: Canadian dollars
GPA: Grade point average
h: hour
KJ: Kylie Johnston
﻿min: minute﻿
OSCE: Objective structured clinical examination
SP: Standardised patient
SPS: ﻿standardised patient scenarios
USD: United states dollars
