# Supplementary material for: Developing physiotherapy student safety skills in readiness for clinical placement using standardised patients compared with peer-role play: a pilot non-randomised controlled trial
Source: BMC Med Educ. 2017 Aug 10;17:133. doi: 10.1186/s12909-017-0973-5 (PMC5553918; doi:10.1186/s12909-017-0973-5)
Supplement: Supplementary file 1 — Feedback checklist and key learning objectives for standardised patient scenario workshop. Description of data: The checklist used by clinical educators during the standardised patient scenario workshops to provide standardised feedback covering the key learning objectives for the workshop. (PDF 291 kb) [file 12909_2017_973_MOESM1_ESM.pdf]

Clinical educator feedback checklist for key learning objectives

Debrief checklist

**Specific Assessments**			
**1. Familiarisation with attachments**			
☐ Identified the four patient attachments prior to commencing mobilisation			
☐ Prepared attachments for mobilisation (e.g. rolled TED's up, untangled IV line/NS, moved IVT in direction of transfer)			
☐ Managed attachments safely and appropriately throughout session			
**2. Planning of mobilisation task**			
**Assessment of patient capacity to complete transfer**			
☐ Checked patient pain control is adequate			
☐ Checked strength	☐ legs	☐ arms	☐ trunk
**Set up of physical environment for safe transfer**			
☐ Suitable bed height			
☐ Bed brakes on			
☐ TED's rolled up or non-slip socks/footwear on			
☐ Walking frame ready and adjusted			
**Communication with patient and assistant**			
☐ Assistant briefed and ready for mobilisation			
☐ Patient informed and consented to mobilisation task			
**3. Actual mobilisation task**			
Safe and effective mobilisation			
☐ Positioned self, and assistant/s close enough to be able to support patient if they lose balance			
☐ Clear instructions to patient and assistant			
☐ Protected patients IV line, catheter, nasal specs whilst transferring and ambulating			
☐ Completed mobility task:	☐ SOEB	☐ SOOB in chair	☐ Ambulation
**4. Reflection of performance**			
☐ Able to identify what was done well			
☐ Able to identify areas for improvement			
☐ Strategies discussed for subsequent task/s where relevant			
☐ Peer review completed			
Overall feedback : Consider both professional behaviour and safety as either satisfactory or unsatisfactory, when the full performance of task has been considered			
**Professional behaviour** (e.g. appearance, introduction to patient)			**☐ satisfactory / ☐ unsatisfactory**
**Safety**			**☐ satisfactory / ☐ unsatisfactory**

Learning objectives

1. **Demonstrate familiarisation** with the patient attachments of IVT, Nasal specs, IDC, TED's
2. **Demonstrate the ability to planning** the necessary procedures to enable the safe and effective transfer / mobilisation task (SOOB-stand-walking) Including:
    - Preparation of physical environment and assessment of patient for safe transfer
    - Communication to reassure patient and explanation planned mobility task
    - Communication to assistant/s to ensure safe and effective mobility task
    - Management of patient attachments safely throughout entire mobility task
3. **Demonstrate safe and effective mobilisation of patient WBAT**
   Practice of procedures involved with assisting patient transfer / mobilisation task (SOOB-stand-walking)
4. **Be able to reflect** on performance during execution of the procedures (self-reflection and peer assessment)
