# Supplementary material for: Developing physiotherapy student safety skills in readiness for clinical placement using standardised patients compared with peer-role play: a pilot non-randomised controlled trial
Source: BMC Med Educ. 2017 Aug 10;17:133. doi: 10.1186/s12909-017-0973-5 (PMC5553918; doi:10.1186/s12909-017-0973-5)
Supplement: Supplementary file 2 — Training information provided to SPs. Description of data: Information provided to the SPs including background information for the patient and the required behaviours, movements and attachments required for the workshop scenarios. (PDF 514 kb) [file 12909_2017_973_MOESM2_ESM.pdf]

SP training session overview

There will be two patient encounters during each of the practical sessions (8-11 am and 2-5pm). There will be five SPs and approximately 40 students. Students will be working in groups of four, with two rotating pairs and each SPs will be required to complete each Encounter twice. The morning and afternoon sessions will follow the same timeframes and Encounters, with the addition of video recording for the afternoon session. All video footage will be deleted following the student debrief.
**** Consent is required for this and we will ask all students /SPs to sign a talent release form****

Encounter 1 will be a straightforward mobility task which required the students to do an abbreviated and basic assessment before getting you out of bed.
Commence with video.

**Audio-visual aids for character development**	***Please review video***

**Scenario details**	Encounter 1
**Patient Name**	Jill Sundance
**Age Range**	70 years old
**Diagnosis**	Day 1 after your fractured right hip (neck of femur / NOF) has been fixed surgically with a dynamic hip screw.
**Patients Story**	**Encounter 1:** You are a 70 year old woman and you were admitted to hospital after you broke your hip yesterday when you were loading your washing machine. You slipped on the lino and fell on to the pile of clothes<br>You were taken to theatre and had surgery on it and it was fixed with a dynamic hip screw yesterday. Your wound is on the outer side of your upper right thigh just below the hip.
**Setting**	Orthopaedic Inpatient Ward – single or shared room
**Background information**	You are retired and live at home with your spouse and a small dog. You still drive and you live in a one storey unit with one step at the front and back entrances. The shower is separate to the bath.<br>You have had good experiences with the health care system in the past. You have a married daughter who lives locally and she is available to help with the groceries and take you to appointments. She has a family of her own and children in high school.
**Physical characteristics**	You are wearing a hospital gown and resting in bed, with the back rest raised by 40 degrees. You are a bit slumped in bed and looking tired and slightly pale.<br>**You will have four attachments:**<br>**1. TED stockings (knee /thigh high).**

	**2. A drip into your hand /elbow (on mobile trolley)**<br>**3. A urinary catheter with a drainage bag anchored to your thigh**<br>**4. Oxygen into your nose (nasal specs)**
**Patient affect/behaviours**	You are alert and co-operative. You are a bit anxious about moving your leg and about getting out of bed so soon after the operation.
**Patients current concerns and expectations**	You are happy you are alive and the surgery was over. However, you are concerned about how would you go moving? You feel unsure about why you need to get up so soon and are very concerned that you may fall over again.
**Visual representation of presentation and attachments**	
	You feel the pain over the wound site which is intermittent and feels deep. You have no pins and needles.
	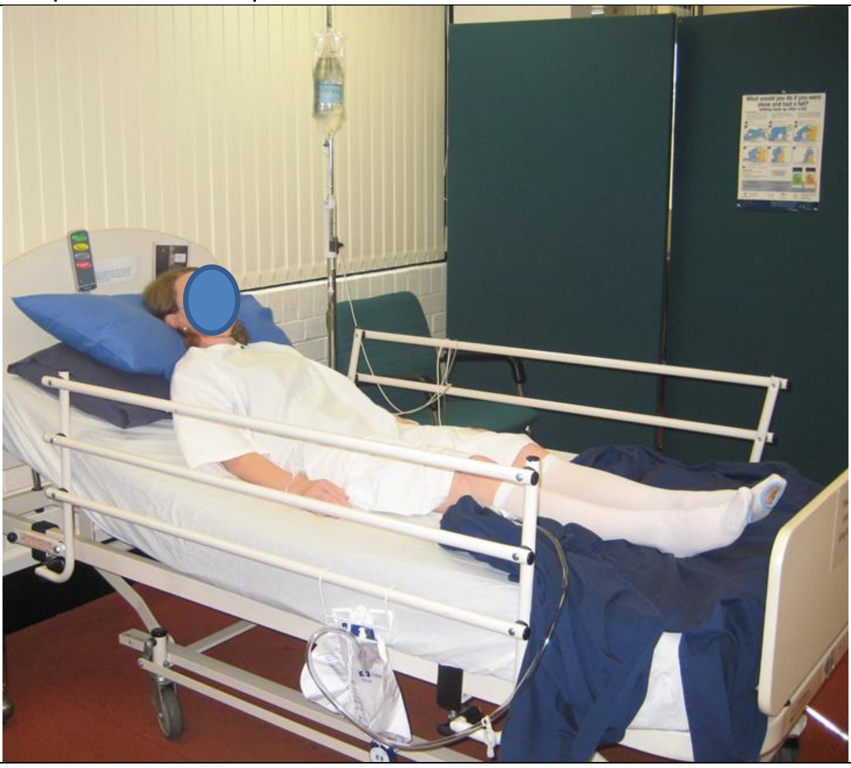
	Location of attachments:<br>1 pair of knee high TED stockings<br>2 IVT /drip into front of left elbow secured with dressing<br>3 Catheter bag anchored to right thigh<br>4 Oxygen into nose (nasal specs)

**Movement limitations/ capabilities**	**Normal movement**<br>You normally walk with a walking stick due to some R knee arthritis and can still get out and about, driving the car. You enjoy socialising at the local seniors club.<br><br>**Movements in bed**<br>*When doing and movements of the leg it will feel tight on the outside of your leg where the wound is (e.g. as you slide your leg on the bed bending your knee (hip and knee flexion).*<br>You haven't bent your hip yet.<br>When you try it will feel like it doesn't belong to you and it is very difficult to achieve much more than about 20 degrees of hip bend.<br>You are able to move your ankles up and down without any problem and have equal strength in them and in your toes<br>Moving your leg out to the side (abduction) – feels heavy and hurts (3/10)<br>Contracting your thigh muscle to straighten your knee (quads) is not painful but it may take one or two tries before you can maintain / hold a good contraction for a few seconds<br>You will find it very hard to lift your R leg up straight and you will need help moving the leg off the bed.<br><br>**Getting out of bed**<br>*You will be anxious but cooperative. You will express concern about moving the leg.*<br>When the head of the bed is raised to 70 degrees it does not hurt but you are anxious about causing pain.<br>Moving from this position to the edge of the bed you will need help from the student to move your leg.<br>When sitting on the edge of the bed you will feel fine, not dizzy for Encounter 1. You will be stood at the edge of the bed, using a walking frame on the first day (see video footage).<br><br>**Walking and sitting out of bed**<br>*You will be anxious but cooperative. You will express concern about falling over.*<br><br>You are allowed to put as much weight as you find comfortable through your operated leg. You will be a worried about this and will not put all of your weight on your operated leg as it is a bit painful (2/10).<br>Once you are standing you will be asked to move your weight from one leg to the other and this will be OK but you will prefer to get off the affected leg more quickly when walking a few steps.<br>You will tend to maintain a stiff R leg when walking. You may be taught to walk in a particular pattern initially to reduce the time and weight through your leg. If you are not taught a specific pattern to walk in you will prefer to spend less time on your affected leg and take shorter

	steps to achieve this.<br>This is the first time you have used a frame and will need clear instructions on how to use it. You can ask the student ***"..how do I use this?"*** Wait for instructions from the student before starting to walk.<br>If you end up sitting out of bed you will want to know "..**how long you will have to stay there?"**<br>Students should leave you with a call bell. So you can call for assistance.
**Medication (Props) Drug charts:**	Your pain will be pretty well covered, as you have had the pain relief - panadol and morphine about half an hour ago.
**Investigations (Props)**	Student vignette which includes a brief summary of your history until now (e.g. surgery, pain relief, prev mobility and no other serious medical problems).

Encounter 2 will be the same patient scenario with added complexity once mobilisation has commenced. The added complexity will include one of the following:

Mrs Sundance will:

1. Report feeling dizzy and unwell once sitting on the edge of the bed.
   If the situation is not addressed (e.g. deep breaths, wriggle toes) this will lead to a sudden fainting episode back on to the bed.
2. Feel very nauseas and need to vomit when sitting on edge of bed or once standing.
   Students will need to provide a vomit bag, encourage deep breaths etc.
3. Report feeling unsteady and a bit wobbly in the legs. If this is not addressed by added physical support by the students, you will become physically wobbly in the legs and on the verge of collapsing after taking one or two steps (but you will be able to catch yourself with the frame, you will NOT need to pretend to or actually fall)

**Scenario details**	**Encounter 2**
**Patient Name**	Jill Sundance
**Age Range**	70 years old
**Diagnosis**	Day 1 after your fractured right hip (neck of femur / NOF) has been fixed surgically with a dynamic hip screw.
**Patients Story**	**Encounter 2:** You are a 70 year old woman and you were admitted to hospital after you broke your hip yesterday when you were loading your washing machine. You slipped on the lino and fell on to the pile of clothes<br>You were taken to theatre and had surgery on it and it was fixed with a dynamic hip screw yesterday. Your wound is on the outer side of your upper right thigh just below the hip.<br>In the past you have reacted badly to anaesthetics which make you unwell (dizzy, light headed and nauseous).
**Setting**	Orthopaedic Inpatient Ward – single or shared room
**Background information**	You are retired and live at home with your spouse and a small dog. You still drive and you live in a one storey unit with one step at the front and back entrances. The shower is separate to the bath.<br>You have had good experiences with the health care system in the past. You have a married daughter who lives locally and she is available to help with the groceries and take you to appointments. She has a family of her own and children in high school.
**Physical characteristics**	You are wearing a hospital gown and resting in bed, with the back rest raised by 40 degrees. You are a bit slumped in bed and looking tired and slightly pale. **You are feeling slightly sick /nauseated / wobbly**<br>**You will have four attachments:**<br>**1. TED stockings (knee /thigh high).**<br>**2. A drip into your hand /elbow (on mobile trolley)**<br>**3. A urinary catheter with a drainage bag anchored to your thigh**<br>**4. Oxygen into your nose (nasal specs)**

**Patient affect/behaviours**	You are alert and co-operative. You are a bit anxious about moving your leg and about getting out of bed so soon after the operation.
**Patients current concerns and expectations**	You are happy you are alive and the surgery was over. However, you are concerned about how would you go moving? You feel unsure about why you need to get up so soon and are very concerned that you may fall over again.
**Visual representation of presentation and attachments**	
	You feel the symptoms in the coloured area. They are intermittent and feel deep when they come on. You have no pins and needles
	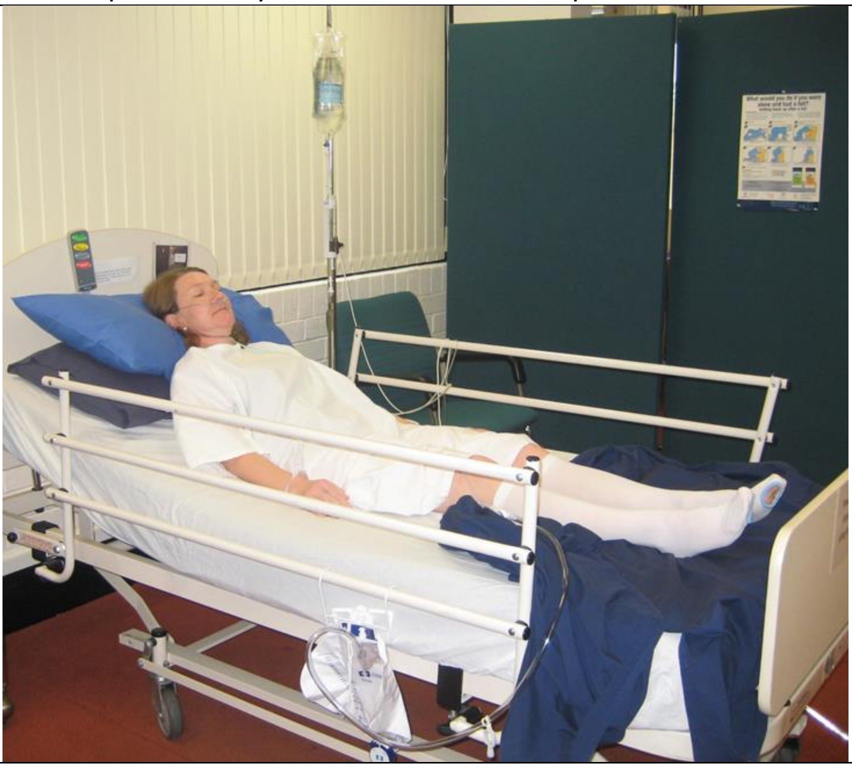
	Location of attachments:<br>1 pair of knee high TED stockings<br>2 IVT /drip into front of left elbow secured with dressing<br>3 Catheter bag anchored to right thigh<br>4 Oxygen into nose (nasal specs)

**Movement limitations/ capabilities**	**Normal movement**<br>You normally walk with a walking stick due to some R knee arthritis and can still get out and about, driving the car. You enjoy socialising at the local seniors club.<br><br>**Movements in bed**<br>*When doing and movements of the leg it will feel tight on the outside of your leg where the wound is (e.g. as you slide your leg on the bed bending your knee (hip and knee flexion).*<br>You haven't bent your hip yet.<br>When you try it will feel like it doesn't belong to you and it is very difficult to achieve much more than about 20 degrees of hip bend.<br>You are able to move your ankles up and down without any problem and have equal strength in them and in your toes<br>Moving your leg out to the side (abduction) – feels heavy and hurts (3/10)<br>Contracting your thigh muscle to straighten your knee (quads) is not painful but it may take one or two tries before you can maintain / hold a good contraction for a few seconds<br>You will find it very hard to lift your R leg up straight and you will need help moving the leg off the bed.<br><br>**Managing attachments**<br>Students will need to identify and manage all attachments during the transfer. You can let the attachments go to the end of their reach and then provide cues such as I can feel something pulling / hurting …<br><br>**Getting out of bed**<br>*You will be anxious but cooperative. You will express concern about moving the leg.*<br>When the head of the bed is raised to 70 degrees it does not hurt but you are anxious about causing pain.<br>Moving from this position to the edge of the bed you will need help from the student to move your leg.<br>When sitting on the edge of the bed you will feel:<br><br>1. **Dizzy and unwell**. If the situation is not addressed (e.g. deep breaths, wriggle toes) within a few seconds, tell them again before **suddenly fainting (safely) back onto the bed**.<br><br>2. **Nauseas and need to vomit** when sitting on edge of bed or once standing. Students will need to provide a vomit bag, encourage deep breaths etc. **If the students do not respond within a few seconds tell them again before: feigning a vomit.**<br><br>3. **Unsteady and a bit wobbly in the legs**. If this situation is not addressed by added physical / verbal support by the students with

	in a few seconds, tell them again before becoming physically wobbly in the legs and on the verge of collapsing after taking one or two steps (but you will be able to catch yourself with the frame, you will NOT need to pretend to or actually fall).<br><br>**Walking and sitting out of bed**<br>*You will be anxious but cooperative. You will express concern about falling over.*<br><br>You are allowed to put as much weight as you find comfortable through your operated leg. You will be a worried about this and will not put all of your weight on your operated leg as it is a bit painful (2/10).<br>Once you are standing you will be asked to move your weight from one leg to the other and this will be OK but you will prefer to get off the affected leg more quickly when walking a few steps.<br>You will tend to maintain a stiff R leg when walking. You may be taught to walk in a particular pattern initially to reduce the time and weight through your leg. If you are not taught a specific pattern to walk in you will prefer to spend less time on your affected leg and take shorter steps to achieve this.<br>This is the first time you have used a frame and will need clear instructions on how to use it.<br>You can ask the student ***"..how do I use this?"*** Wait for instructions from the student before starting to walk.<br>You may become wobbly in the legs (as directed by the facilitator?) to add complexity in this scenario.<br>If you end up sitting out of bed you will want to know "..**how long you will have to stay there?"**<br>Students should leave you with a call bell, so you can call for assistance.
**Medication (Props) Drug charts:**	Your pain will be pretty well covered, as you have had the pain relief - panadol and morphine about half an hour ago.
**Investigations (Props)**	Student vignette which includes a brief summary of your history until now (e.g. surgery, pain relief, prev mobility and no other serious medical problems).

Standardised patient (SP) feedback to students

Below is from Blackstock FC, Watson KM, Morris NR, Jones A, Wright A, McMeeken JM, et al. Simulation can contribute a part of cardiorespiratory physiotherapy clinical education: two randomized trials, Simulation in Healthcare: The Journal of The Society for Medical Simulation, 2013;8(1):32-42.

Please feedback to the student/s your responses to these questions in 1 or 2 sentences:

Communication
Did the student ask about your concerns and how well you are able to move?
Did the student explain to you how the session would proceed and how you could assist with the movement?
Could you understand what they wanted you to do?
Did the student seem to care about you?
Did the student listen to what you had to say?

Physical care
Did the student put you at ease during the session?
Did the student handle your body with care and consideration?
Did the student take care of your physical safety?

Professionalism
Did the student look professional?
Did the student act in a manner that was professional?
