# Supplementary material for: Developing physiotherapy student safety skills in readiness for clinical placement using standardised patients compared with peer-role play: a pilot non-randomised controlled trial
Source: BMC Med Educ. 2017 Aug 10;17:133. doi: 10.1186/s12909-017-0973-5 (PMC5553918; doi:10.1186/s12909-017-0973-5)
Supplement: Supplementary file 3 — Timetable for standardised patient scenario workshop. Description of data: The timetable used for the standardised patient scenario workshop allowing each group of students to experience two patient encounters with preparation and debriefing time for each encounter. (PDF 223 kb) [file 12909_2017_973_MOESM3_ESM.pdf]

Additional File 3 Timetable for standardised patient scenarios workshop 2015

	**SP 1 Educator A**	**Debrief Self/peer**	**SP 2 Educator B**	**Debrief Self/peer**	**SP 3 Educator C**	**Debrief Self/peer**	**SP 4 Educator D**	**Debrief Self/peer**	**SP 5 Educator E**	**Debrief Self/peer**
8	intro, pre-workshop questionnaire and prep for all including in C7 20/21 (30 mins)									
830	1A & 2A		1B & 2B		1C & 2C		1D & 2D		1E & 2E	
845	3A & 4A		3B & 4B		3C & 4C		3D & 4D		3E & 4E	
9	5A & 6A	1st feedback 1A,2A,3A,4A	5B & 6B	1st debrief 1B,2B,3B,4B	5C & 6C	1st debrief 1C,2C,3C,4C	5D & 6D	1st debrief 1D,2D,3D,4D	5E & 6E	1st debrief 1E,2E,3E,4E
915	7A & 8A		7B & 8B		7C & 8C		7D & 8D		7E & 8E	
930*	4A & 3A	1st feedback 5A,6A,7A,8A	4B & 3B	1st debrief 5B,6B,7B, 8B	4C & 3C	1st debrief 5C,6C,7C,8C	4D & 3D	1st debrief 5D,6D,7D,8D	4E & 3E	1st debrief 5E,6E,7E,8E
945	2A & 1A		2B & 1B		2C & 1C		2D & 1D		2E & 1E	
10	8A & 7A	Final feedback, questionnaire 1A,2A,3A,4A	8B & 7B	Final debrief, questionnaire 1B,2B,3B,4B	8C & 7C	Final debrief, questionnaire 1C,2C,3C,4C	8D & 7D	Final debrief, questionnaire 1D,2D,3D,4D	8E & 7E	Final debrief, questionnaire 1E,2E,3E,4E
1015	6A & 5A		6B & 5B		6C & 5C		6D & 5D		6E & 5E	
1030		Final feedback, questionnaire 5A,6A,7A,8A		Final debrief, questionnaire 5A,6B,7B,8B		Final debrief, questionnaire 5C,6C,7C,8C		Final debrief, questionnaire 5D,6D,7D,8D		Final debrief, questionnaire 5E,6E,7E,8E
1045										

	SP 1 Educator A	Debrief Self/peer	SP 2 Educator B	Debrief Self/peer	SP 3 Educator C	Debrief Self/peer	SP 4 Educator D	Debrief Self/peer	SP 5 Educator E	Debrief Self/peer
2:00	intro, pre-workshop questionnaire and prep for all including in C7 20/21 (30 mins)									
2:30	1A & 2A		1B & 2B		1C & 2C		1D & 2D		1E & 2E	
2:45	3A & 4A		3B & 4B		3C & 4C		3D & 4D		3E & 4E	
3:00	5A & 6A	1st feedback 1A,2A,3A,4A	5B & 6B	1st debrief 1B,2B,3B,4B	5C & 6C	1st debrief 1C,2C,3C,4C	5D & 6D	1st debrief 1D,2D,3D,4D	5E & 6E	1st debrief 1E,2E,3E,4E
3:15	7A & 8A		7B & 8B		7C & 8C		7D & 8D		7E & 8E	
3:30*	4A & 3A	1st feedback 5A,6A,7A,8A	4B & 3B	1st debrief 5B,6B,7B, 8B	4C & 3C	1st debrief 5C,6C,7C,8C	4D & 3D	1st debrief 5D,6D,7D,8D	4E & 3E	1st debrief 5E,6E,7E,8E
3:45	2A & 1A		2B & 1B		2C & 1C		2D & 1D		2E & 1E	
4:00	8A & 7A	Final feedback, questionnaire 1A,2A,3A,4A	8B & 7B	Final debrief, questionnaire 1B,2B,3B,4B	8C & 7C	Final debrief, questionnaire 1C,2C,3C,4C	8D & 7D	Final debrief, questionnaire 1D,2D,3D,4D	8E & 7E	Final debrief, questionnaire 1E,2E,3E,4E
4:15	6A & 5A		6B & 5B		6C & 5C		6D & 5D		6E & 5E	
4:30		Final feedback, questionnaire 5A,6A,7A,8A		Final debrief, questionnaire 5A,6B,7B,8B		Final debrief, questionnaire 5C,6C,7C,8C		Final debrief, questionnaire 5D,6D,7D,8D		Final debrief, questionnaire 5E,6E,7E,8E
4:45										

***To enable an element of surprise in Encounter 2 the patient will become dizzy / nauseous / wobbly when SOEB, and if not addressed will “faint” safely backwards onto bed / feign vomiting / become wobbly and unsteady on feet but will not fall**

SP standardised patient
