# Supplementary material for: Developing physiotherapy student safety skills in readiness for clinical placement using standardised patients compared with peer-role play: a pilot non-randomised controlled trial
Source: BMC Med Educ. 2017 Aug 10;17:133. doi: 10.1186/s12909-017-0973-5 (PMC5553918; doi:10.1186/s12909-017-0973-5)
Supplement: Supplementary file 4 — Facilitator guide for student debriefing session. Description of data: The guide provided to facilitators to assist the two debriefing sessions immediately following the completion of each of the student encounters with the standardised patient scenarios. (PDF 196 kb) [file 12909_2017_973_MOESM4_ESM.pdf]

Facilitator guide for student debriefing session

Please ensure that each group of four students are comfortably seated in a circle. Then ask the group to talk about:

**Self-reflection**	1. How they thought their session went?<br>2. What worked well for their pair?<br>3. What they would have liked to do differently?<br>4. What would they change for next time?
**Peer feedback**	5. Briefly discuss 3 key areas with each other from the completed peer feedback checklist.<br>6. Watch relevant sections of the videos related to self and peer reflection.

Additional strategies to assist the student/s to debrief:

Reflection of performance - ask students to reflect on each of the learning goals

How did you go with the:

- Familiarisation with attachments – identifying / preparing / managing the attachments (safely and appropriately)?
- Actual mobilisation task- was it safe and effective?
- Planning of the mobilisation task- assessing the patient's capacity to transfer / pain control / limb strength (UL, LL, Grip strength).
- Set up of physical environment for safe transfer- bed height / brakes / TED's/ gait aids
- Communication with patient and assistant- assistant briefing / patient consent
- Reflection of performance – what went well / areas for improvement / strategies for future tasks / peer review

Reflection regarding the feedback from the patient

- How will your performance change following the feedback from the patient about your communication / explanation of the session?
- How will your performance change following the feedback from the patient about your handling during the session?
- How will your performance change following the feedback from the patient about your professional behaviour?
