# Supplementary material for: Developing physiotherapy student safety skills in readiness for clinical placement using standardised patients compared with peer-role play: a pilot non-randomised controlled trial
Source: BMC Med Educ. 2017 Aug 10;17:133. doi: 10.1186/s12909-017-0973-5 (PMC5553918; doi:10.1186/s12909-017-0973-5)
Supplement: Supplementary file 5 — Student vignette. Description of data: The information provided to students prior to the standardised patient scenario workshops which included a summary of all relevant patient information and images of the patient’s attachments to assist in the preparation for the patient encounter. (PDF 312 kb) [file 12909_2017_973_MOESM5_ESM.pdf]

SUNDANCE Jill
DOB 21-8-1940
3 Orange Drive Fullarton SA 5063
Consultant: Dr Riggs

75 year old female admitted to the Fullarton Hospital on 12-5-15 following a fall at home sustaining a fractured right neck of femur (NOF).

Surgical management: 12-5-15 open reduction and internal fixation (ORIF) with a dynamic hip screw.
Post-op orders: weight bearing as tolerated (WBAT)

PMHx: no serious health problems, no previous falls.

Previous mobility: independent using a walking stick.

Post op progress: observations have been stable and her pain has been managed with oral tablets which she last took 30 minutes ago and will be at their peak effectiveness now.

Attachments: (pictures of the patient attachments are provided over the page)

- 2L/min oxygen via nasal prongs
- Indwelling urinary catheter (IDC)
- Intravenous line (IV)inserted into elbow (cubital fossa)
- Thrombo-embolic deterrent stockings (TED)

Patient attachments

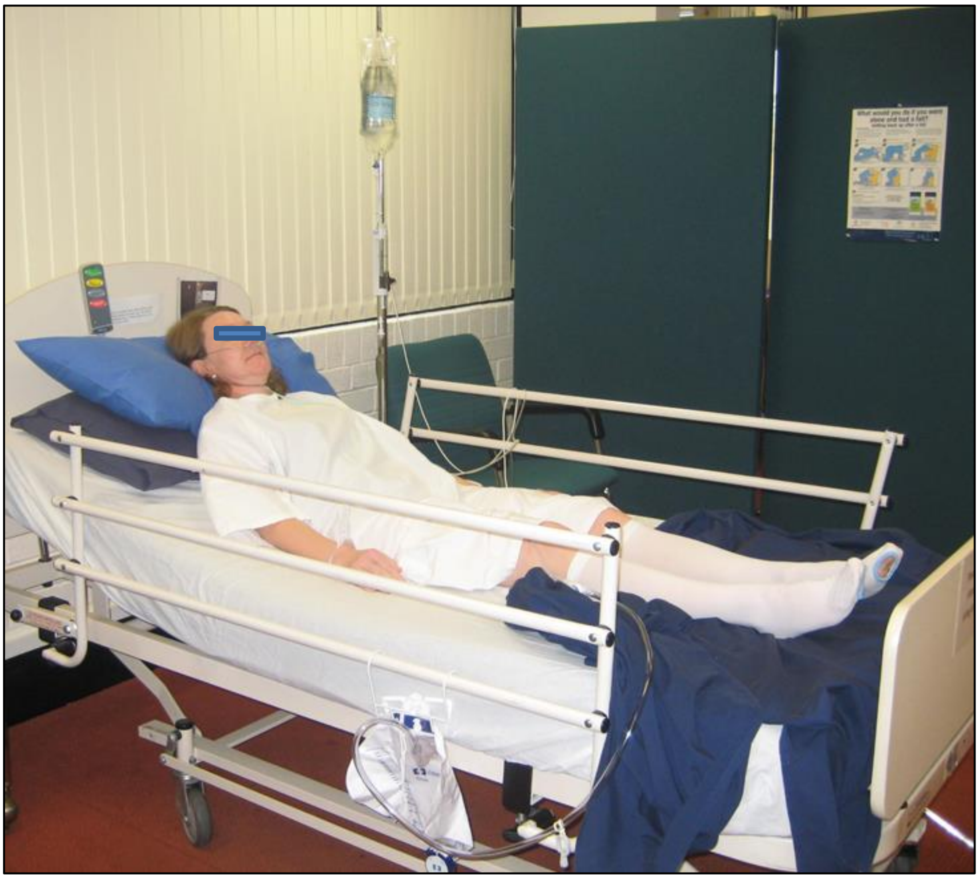

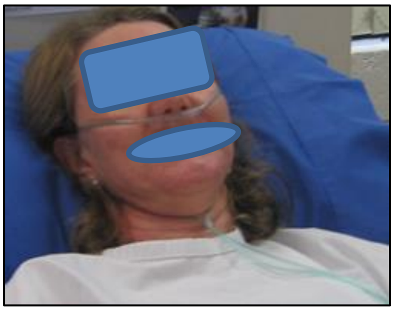

Nasal prongs

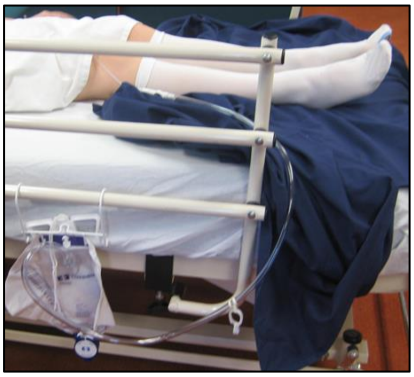

IDC and TED

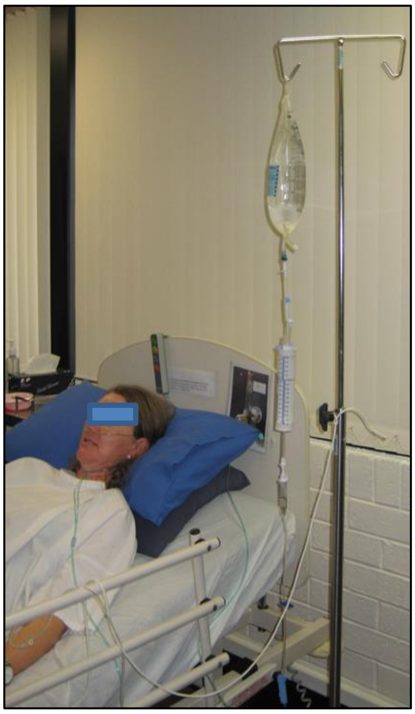

IV
