# Supplementary material for: Developing physiotherapy student safety skills in readiness for clinical placement using standardised patients compared with peer-role play: a pilot non-randomised controlled trial
Source: BMC Med Educ. 2017 Aug 10;17:133. doi: 10.1186/s12909-017-0973-5 (PMC5553918; doi:10.1186/s12909-017-0973-5)
Supplement: Supplementary file 6 — Student response provided for how the standardised patient scenarios could be improved. Description of data: A summary of all verbatim responses provided by students following the completion of the standardised patient scenario workshops in response to the question “How could this experience be improved?” (PDF 212 kb) [file 12909_2017_973_MOESM6_ESM.pdf]

Verbatim student responses

How could this experience be improved?

- perhaps get longer-like 1/2 hour each to perform the whole transfer not just half
- More time / scenarios
- Do more of it.
- I was lucky to only have 2 people (including myself) in my group and we found it very beneficial to have the full time without any interruptions
- maybe more direction early on. But overall very good!
- perhaps a better understanding of requirements and what to expect
- more sessions with different client types. Less waiting around between practice
- maybe we could get constant supervision in a single room without other groups
- more practise with some different patient scenarios
- Would like it to be a week later so we could be a but more prepared. I feel like I haven't learned everything properly yet! Would be good to have feedback on what the right thing to do in our situations.
- increase the time for feedback with the physiotherapist that supervised the session
- more time and opportunities to do this, more time for feedback from teaching support supervising the transfer
- allow more time for preparation
- having more than 1 session
- maybe more variety in patients to practice on
- perhaps a tip to review manual handling techniques before
- preparation prac prior to session
- variety of conditions. Work through entire performance at once rather than in two parts (physio /assistant)
- more opportunities to practice
- Give us a better picture of what the attachments are and how to move them before the simulation, so we can focus on clear explanations and planning for the patient. Everything all at once is too overwhelming and squashes the confidence before we even begun. Or give us more time to faf around with attachments as well as doing the transfer.
- even more feedback, perhaps more specific to the individual - however I understand this is difficult.
- it was excellent!!
- excellent experience, doesn't need improvement
- greater access to actors, ie to repeat it.

- Loved it. Maybe more scenarios with patients.
- little more explanation on how we will be switching roles (no need for introduction etc.)
- give us more chance to do it
- increasing number of tried we get
- more opportunities to do it
- placed in a real /well replicated hospital environment
- Have actual feedback and maybe how to actual tackle scenario
- more interactions and sharing between groups
- different patient used for the second visit. The second group wasn't there to view the first groups attempt so that they could see how they progress
- larger space (something like the nursing labs here at unisa would be ideal)
- done more often! Sometimes the physio paused the scenario for a bit too long
- I thought it was great
- different scenarios, allow us to adapt the next time we did the activity
- Frequency- just more of it.
- Only by getting the experience more often.
- give us the opportunity to do it more frequently!
- being able to do it again would be good so there is time to improve
- more time as therapist
- Nothing really! Maybe a bit more knowledge about attachments prior to the day.
- perhaps more opportunities to role play more than twice
- I think it was great overall-maybe more practice scenarios like this would be helpful.
- more sessions
- prior recap of needed skills just because we did learn it over a month ago.
- definitely
- need more time for each group

Categories	**Comments**
**PATIENT INTERACTIONS**	**19**
Greater frequency	12
More time as therapist	1
More time in scenarios	5
Less waiting / pauses	2
**CHANGES TO INTERACTION**	**12**
Different environment	2
Different patient conditions	3
Alternative scenarios	4
None	3
**CLINICAL EDUCATOR**	**9**
More supervision	2
More feedback	5
Less pauses	1
Direction during patient transfer	1
**PREPARATION**	**8**
Prior to intervention	4
Prior to patient interaction	1
Guidance regarding what to prepare	3
